# Advancing insights in critical COVID-19: unraveling lymphopenia through propensity score matching - Findings from the Multicenter LYMPH-COVID Study

**DOI:** 10.62675/2965-2774.20240236-en

**Published:** 2024-09-18

**Authors:** José Pedro Cidade, Vicente Cês Souza-Dantas, Rafaela Braga Mamfrim, Renata Carnevale Miranda, Henrique Tommasi Caroli, Natália Almeida Oliveira, Alessandra e Figueiredo Thompson, Gabriela E. Oliveira, Pedro Póvoa

**Affiliations:** 1 Department of Intensive Care Hospital São Francisco Xavier Centro Hospitalar Lisboa Ocidental Lisbon Portugal Intensive Care Unit 4, Department of Intensive Care, Hospital São Francisco Xavier, Centro Hospitalar Lisboa Ocidental - Lisbon, Portugal.; 2 Instituto D’Or de Pesquisa e Ensino Rio de Janeiro RJ Brazil Instituto D’Or de Pesquisa e Ensino - Rio de Janeiro (RJ), Brazil.; 3 Hospital Copa D’Or Rio de Janeiro RJ Brazil Hospital Copa D’Or - Rio de Janeiro (RJ), Brazil.

**Keywords:** Lymphopenia, COVID-19, Coronavirus infections, SARS-CoV-2, Mortality, Critical care, Intensive care units

## Abstract

**Objective:**

To elucidate the impact of lymphopenia on critical COVID-19 patient outcomes.

**Methods:**

We conducted a multicenter prospective cohort study across five hospitals in Portugal and Brazil from 2020 to 2021. The study included adult patients admitted to the intensive care unit with SARS-CoV-2 pneumonia. Patients were categorized into two groups based on their lymphocyte counts within 48 hours of intensive care unit admission: the Lymphopenia Group (lymphocyte serum count < 1 × 10^9^/L) and the Nonlymphopenia Group. Multivariate logistic regression, propensity score matching, Kaplan‒Meier survival curve analysis and Cox proportional hazards regression analysis were used.

**Results:**

A total of 912 patients were enrolled, with 191 (20.9%) in the Nonlymphopenia Group and 721 (79.1%) in the Lymphopenia Group. Lymphopenia patients displayed significantly elevated disease severity indices, including Sequential Organ Failure Assessment and Simplified Acute Physiology Score 3 scores, at intensive care unit admission (p = 0.001 and p < 0.001, respectively). Additionally, they presented heightened requirements for vasopressor support (p = 0.045) and prolonged intensive care unit and in-hospital stays (both p < 0.001). Multivariate logistic regression analysis after propensity score matching revealed a significant contribution of lymphopenia to mortality, with an odds ratio of 1,621 (95%CI: 1,275 - 2,048; p < 0.001). Interaction models revealed an increase of 8% in mortality for each decade of longevity in patients with concomitant lymphopenia. In the subanalysis utilizing three-group stratification, the Severe Lymphopenia Group had the highest mortality rate, not only in direct comparisons but also in Kaplan‒Meier survival analysis (log-rank test p = 0.0048).

**Conclusion:**

Lymphopenia in COVID-19 patients is associated with increased disease severity and an increased risk of mortality, underscoring the need for prompt support for critically ill high-risk patients. These findings offer important insights into improving patient care strategies for COVID-19 patients.

## INTRODUCTION

Lymphopenia is a distinctive feature observed in critically ill coronavirus disease 2019 (COVID-19) patients and purportedly serves as a definitive hallmark of the disease.^( 1 )^ Over the past few years, it has been a marker of interest, motivating researchers to find compelling evidence that supports its potential as a specific indicator linked to disease severity and high mortality rates.^( 2 - 5 )^ The incidence of lymphopenia among COVID-19 patients admitted to the hospital has been reported to be 63%, increasing to nearly 85% among those in critical condition.^( 6 )^ This underscores its unique position as a strong link between the dysregulated inflammatory response and cytokine production described in the disease pathophysiology.^( 7 , 8 )^ Unraveling the intricate mechanisms underlying lymphopenia in COVID-19 patients may reveal a new direct influence on the immune system and the outcomes of COVID-19 patients.

Multiple clinical and laboratory biomarkers have been extensively characterized, evaluating their predictive value in the prompt identification of patients needing multiorgan support and those facing a high risk of mortality.^( 9 , 10 )^ Lymphopenia induced by severe acute respiratory syndrome coronavirus 2 (SARS-CoV-2), which is attributed to the infection and replication of the virus in immune cells, culminates in apoptosis and is strongly correlated with the need for intensive care unit (ICU) admission and poor outcomes.^( 11 - 15 )^ Furthermore, recent evidence has shown that lymphopenia (defined as an absolute serum lymphocyte count < 1.0 × 10^9^/L) upon ICU admission is significantly associated with increased requirements for mechanical ventilation, renal replacement therapies, and vasopressor support.^( 3 , 16 - 19 )^ Ultimately, a recent meta-analysis has positioned lymphopenia as an essential evaluative biomarker, indicating its utility as a prognostic marker in COVID-19 patients, particularly among younger individuals. This association led to a threefold heightened risk of unfavorable outcomes in hospitalized COVID-19 patients in another study.^( 6 )^

However, evidence is still lacking concerning whether the observed association between lymphopenia and mortality stems directly from the lymphopenia itself or primarily reflects the heightened severity of the underlying COVID-19 infection. While substantial evidence has indicated a robust correlation between lymphopenia and adverse outcomes, it is imperative to acknowledge the existing need to elucidate the independent impact of lymphopenia on mortality risk within the context of COVID-19 and its true utility as a prognostic target in this disease.^( 20 - 22 )^ To address this issue, our study aimed to evaluate the impact of lymphopenia on critical COVID-19 patient outcomes via a propensity score matching approach.

## METHODS

### Study design and cohort definition

We performed a prospective multicenter observational cohort study in five ICUs in Brazil and Portugal. The Ethics Committees of the Hospital CopaStar and Hospital Copa D’Or in Rio de Janeiro, Brazil, and the Portuguese Ethics Committee for Clinical Investigation in Lisbon approved this study (CAAE: 17079119.7.0000.5249; and REC: 2020_EO_02, respectively).

All adult patients admitted consecutively to the ICU between January 1st, 2020, and March 31, 2021, were considered for the study. Patients were included if they were admitted to the ICU with a COVID-19 respiratory infection diagnosis and a length of stay of at least 72 hours. COVID-19 respiratory infection was diagnosed via clinical and radiological criteria confirming pulmonary involvement along with a SARS-CoV-2-positive reverse transcription polymerase chain reaction (RT‒PCR) test.

Within the study cohort, patients were stratified into two distinct groups on the basis of their lymphocyte counts within 48 hours of ICU admission: the Lymphopenia Group, comprising all patients with documented lymphopenia (absolute lymphocyte serum count < 1 × 10^9^/L), and the Nonlymphopenia Group, which included all patients without a diagnosis of lymphopenia.

In a different subanalysis, patients were further stratified into three distinct groups (three-group stratification) to evaluate the impact of the degree of lymphopenia on the primary outcome: the Severe Lymphopenia Group (absolute lymphocyte serum count ≤ 0.5 × 10^9^/L), the Non-Severe Lymphopenia Group (absolute lymphocyte serum count > 0.5 × 10^9^/L and < 1 × 10^9^/L) and the Nonlymphopenia Group (absolute lymphocyte serum count ≥ 1 × 10^9^/L).

### Data collection and management

Clinical data were prospectively collected from the patients’ electronic health records and included demographics (age and sex), comorbidities (obesity, smoking status, hypertension, diabetes, chronic obstructive pulmonary disease [COPD], coronary heart disease, chronic kidney disease [CKD], and malignancy), daily laboratory values (absolute serum lymphocyte count, C-reactive protein [CRP], procalcitonin [PCT] and troponin), Simplified Acute Physiology Score III (SAPS III), Sequential Organ Failure Assessment (SOFA) score, organ support requirements (mechanical ventilation, vasopressor support and renal replacement therapy), and outcome data (ICU and in-hospital lengths of stay and mortality rates). The data were stored in a pseudoanonymized database.

The primary outcome was the all-cause ICU mortality rate. As secondary outcomes, the ICU and in-hospital length of stay and organ support requirements, including respiratory, hemodynamic or renal support, were used.

### Statistical analysis plan

#### Descriptive statistics and univariate analysis

In the analyzed population, univariate and multivariate analyses were performed to compare the unmatched Lymphopenia and Nonlymphopenia Groups. Continuous variables are expressed as the mean and standard deviation (SD) for Gaussian distributions and as median (interquartile range [IQR]) for nonnormally distributed variables. Categorical variables are presented as numbers and percentages. Univariate analysis was performed via Student’s t test and the Mann‒Whitney U test for continuous variables and the χ2 test for categorical variables.

#### Survival analysis

For survival investigation, Kaplan‒Meier survival curves were generated to assess 28-day mortality rates, complemented with the respective log-rank test. Patients who were discharged or transferred were considered censored observations in the analysis, ensuring a comprehensive analysis of the time-to-event outcomes. We determined that the occurrence of competing risks was minimal for this analysis. Cox regression models for the primary outcome were produced, accounting for the covariables found to be significantly associated with mortality during the univariate analysis. The variables included were age, sex, and comorbidities (obesity, hypertension, diabetes, COPD, chronic heart disease, and CKD) and the SAPS II and SOFA scores at ICU admission.

#### Multivariate logistic regression analysis

A multivariable logistic regression was conducted to comprehensively examine the influence of key variables on the primary outcome. The significance threshold for variable inclusion in the model was set at p < 0.05, ensuring a rigorous selection criterion. The variables incorporated into the model included age, sex, the presence of lymphopenia at ICU admission, comorbidities, and the SAPS II and SOFA scores at ICU admission.

Moreover, interaction models were systematically constructed to estimate the potential cumulative impacts of the independent variables. Following the results obtained in our preceding analysis, where lymphopenia, age, and SOFA score at admission exhibited significant associations, we specifically explored interactions among these variables. The selection of the best model for comparison was determined by identifying the model with the lowest Akaike information criterion (AIC).

#### Propensity score matching

To create comparable Nonlymphopenia and Lymphopenia study groups, optimal neighbor 1-to-1 propensity score matching was employed. Propensity scores were derived through logistic regression modeling, incorporating 10 matching criteria—age, sex, obesity, hypertension, diabetes, COPD, chronic heart disease, CKD, and SAPS II and SOFA scores at ICU admission—using the MatchIt package in R. Calipers, with a specified width of 0.05, were applied during propensity score matching to enhance balance between groups.

To evaluate the effectiveness of the matching procedure, balance diagnostics, including love plots, propensity score distribution plots, and standardized mean differences, were thoroughly examined. The standardized mean differences were calculated to assess the balance achieved after matching, ensuring that the matched groups were comparable in terms of baseline covariates.

#### Handling of missing data

Patients whose data exceeded 5% of the analyzed variables or whose absolute serum lymphocyte count at ICU admission was missing were excluded from the analysis; a conservative approach was used to maintain data integrity and prevent potential bias. Missing data below this rate were imputed using variables’ mean values to minimize systematic bias and maintain the representativeness of the dataset. This approach allowed us to preserve the internal validity of our analyses.

All calculations were performed via the Statistical Package for the Social Sciences (SPSS) interface, version 26.0.0.0 and R, version 4.0.3. p values < 0.05 were considered statistically significant.

## RESULTS

**Figure 1:**
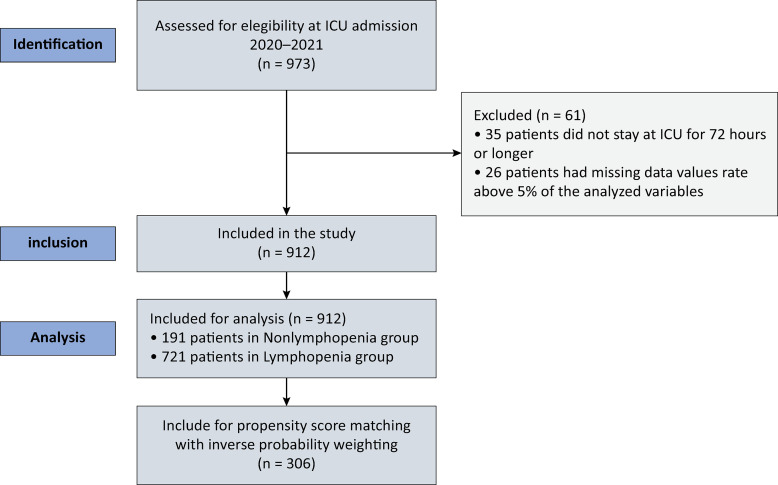
Selection of the participants in the study.

After identification, 973 patients were initially eligible for the study. Among these patients, 61 patients were excluded from the statistical analysis, 35 patients were excluded because they did not stay in the ICU for 72 hours or longer, and 26 patients were excluded because more than 5% of the analyzed variables were missing data. The remaining 912 patients were included, as depicted in figure 1 . One hundred ninety-one patients (20.9%) were included in the Nonlymphopenia Group, and 721 patients (79.1%) presented with lymphopenia at admission with an absolute lymphocyte serum count < 1 × 10^9^/L. The patients’ baseline demographic and primary clinical characteristics are summarized in table 1 .

**Table 1:** Demographics, primary clinical characteristics and outcomes in the Nonlymphopenia and Lymphopenia Groups

	Nonlymphopenia Group (n = 191; 20.9%)	Lymphopenia Group (n = 721; 79.1%)	p valor
Age (years)	60.7 ± 17.9	66.6 ± 15.4	< 0.001
Sex (males)	108 (56.5)	484 (67.1)	0.004
Comorbidities			
COPD	5 (2.6)	57 (7.9)	0.067
Asthma	16 (8.4)	29 (4)	0.058
Chronic kidney disease	13 (6.8)	62 (8.6)	0.12
Obesity	37 (19.4)	110 (15.3)	0.486
Diabetes Mellitus	40 (20.9)	200 (27.7)	0.145
Heart disease	31 (16.2)	172 (23.9)	0.001
ICU admission			
C-reactive protein (mg/dL)	6.4 (2.9 - 14.9)	7.4 (4.7 - 16.5)	0.005
Procalcitonin (ng/mL)	0.1 (0.07 - 0.17)	0.12 (0.08 - 0.29)	0.005
SOFA at admission	1 (0 - 2)	4 (2 - 5)	< 0.001
SAPS III at admission	45.8 ± 13.1	49.5 ± 12.2	< 0.001
Laboratory results			
Max registered C-reactive protein (mg/dL)	8.3 (3.4 - 19.6)	15.5 (6.7 - 23.1)	< 0.001
Max registered procalcitonin (ng/mL)	0.46 (0.14 - 0.77)	0.5 (0.17 - 0.9)	0.286
Minimum leucocytes count registered (x10^9^)	6.8 ± 3.7	5.32 ± 2.4	< 0.001
Max troponin registered (ng/mL)	25 (8.5 - 56.7)	38 (13 - 111)	0.099
Outcomes			
Mechanical ventilation	42 (22)	199 (27.6)	0.229
Vasopressor support	38 (19.9)	195 (27)	0.045
Renal replacement therapy	16 (8.4)	96 (13.3)	0.054
ICU length of stay (days)	5 (3 - 10)	9 (5 - 16.5)	< 0.001
Hospital length of stay (days)	8 (3 ± 14)	10 (6 ± 22)	< 0.001
Mortality	17 (8.9)	79 (11)	0.621

COPD - chronic obstructive pulmonary disease; ICU - intensive care unit; SOFA - Sequential Organ Failure Assessment; SAPS - Simplified Acute Physiology Score. Results expressed as the mean ± standard deviation, n (%) or median (interquartile range).

Patients in the Lymphopenia Group were significantly older and more likely to be males than those in the Nonlymphopenia Group were. These patients also presented with higher SOFA and SAPS III scores at ICU admission (p < 0.001 for both analyses) and significantly higher serum inflammatory biomarker levels (CRP and PCT) at both admission and during their ICU stay. Furthermore, patients who presented with lymphopenia had a higher rate of requirement for vasopressor support, although the groups did not differ in terms of respiratory or renal replacement therapies.

With respect to the outcome analysis, the ICU mortality rates did not differ between the groups. This result was also supported by the Kaplan–Meier survival analysis of both groups, with a log-rank test of p = 0.092 ( Figure 1S - Supplementary Material ). However, the patients in the Lymphopenia Group remained in both the ICU and the hospital longer (p < 0.001, in both analyses). Consistently, the Cox regression model, incorporating variables significantly associated with mortality, demonstrated no statistically significant association between the Lymphopenia Group and mortality (hazard ratio = 1.0448, 95%CI: 0.5133 - 2.127), as illustrated in table 1S ( Supplementary Material ).

Multivariate logistic regression analysis revealed a significant risk contribution of lymphopenia to mortality, with an odds ratio of 1.110 (95%CI: 1,022 - 1,206; p = 0.011), concomitant with age, SOFA score at ICU admission, and previous medical history of COPD and CKD, as depicted in table 2 . Furthermore, interaction models revealed a significant association between age and lymphopenia, with an increase of 8% in mortality for each decade of longevity in patients with concomitant lymphopenia (odds ratio 1.080, p = 0.025) ( Table 2S, Supplementary Material ). The linearity of the interaction coefficient was confirmed by the Box–Tidwell test (-7.55x10^-6^, p = 0.99). No significant interaction was found between lymphopenia and SOFA score at admission (p = 0.17), previous history of COPD (p = 0.32) or previous history of CKD (p = 0.07), although the estimated coefficient suggests a potential trend toward increased mortality risk among patients with both lymphopenia and higher SOFA scores.

**Table 2:** Multivariable logistic regression results for estimation of potential cumulative impacts of independent variables on mortality*

	Odds ratio	95%CI	p value
Age	1.070	1.043 - 1.098	< 0.001
Sex (male)	1. 147	0.768 - 2.159	0.601
SAPS III at ICU admission	1.008	0.984 - 1.032	0.493
SOFA score at admission	1.248	1.161 - 1.340	< 0.001
Lymphopenia	1.110	1.022 - 1.206	0.011
Chronic obstructive pulmonary disease	2.160	1.032 - 4.524	0.043
Chronic kidney disease	3.385	1.881 - 6.104	< 0.001
Obesity	1.808	0.960 - 3.404	0.079
Diabetes Mellitus	0.764	0.481 - 1.212	0.315
Chronic heart failure	1.299	0.762 - 2.215	0.313

95%CI - 95% confidence interval; SAPS - Simplified Acute Physiology Score; ICU - intensive care unit; SOFA - Sequential Organ Failure Assessment. * Akaike Information Criterion 501.

**Figure 2:**
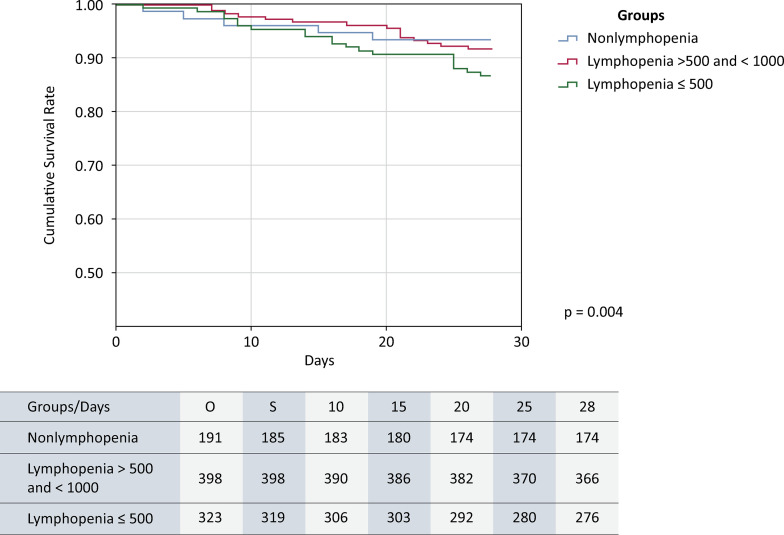
Kaplan-Meier survival analysis in the subanalysis utilizing three-group stratification (Nonlymphopenia Group, Nonsevere Lymphopenia Group and Severe Lymphopenia Group).

Three hundred and six patients were matched between the groups via propensity score matching while the previously stated variables were considered for matching. The main demographic characteristics and propensity score distribution balance analysis (using standard deviation calculations, love plots and distribution plots) were included in the Supplementary Material ( Tables 3S and 4S and Figures 2S and 3S ). Multivariable logistic regression analysis after matching revealed a significant risk contribution of lymphopenia to mortality, with a higher odds ratio of 1.621 (95%CI: 1.275 - 2.048; p < 0.001) with the proposed covariables in the proposed model ( Table 3 ). Kaplan–Meier survival analysis did not reveal a difference in survival over time between the matched Lymphopenia and Nonlymphopenia Groups (log-rank test p = 0.48) ( Figure 4S - Supplementary Material ).

**Table 3:** Multivariable logistic regression results for estimation of potential cumulative impacts of independent variables on mortality after propensity score matching*

	Odds ratio	95%CI	p value
Age	1.035	1.010 - 1.060	0.005
Sex (male)	0.285	0.167 - 0.485	< 0.001
SAPS III at ICU admission	0.998	0.976 - 1.021	0.914
SOFA score at admission	1.438	1.290 - 1.608	< 0.001
Lymphopenia	1.621	1.275 - 2.048	<0.001
Chronic obstructive pulmonary disease	2.214	0.853 - 5.753	0.112
Chronic kidney disease	8.190	3.166 - 7.215	< 0.001
Obesity	2.706	1.839 - 3.594	< 0.001
Diabetes Mellitus	1.955	0.905 - 2.235	0.08
Chronic heart failure	1.006	0.946 - 1.069	0.295

95%CI - 95% confidence interval; SAPS - Simplified Acute Physiology Score; ICU - intensive care unit; SOFA - Sequential Organ Failure Assessment. * Akaike Information Criterion 408.

In the subanalysis utilizing three-group stratification, the Severe Lymphopenia Group presented significantly higher organ dysfunction scores upon ICU admission, increased organ support requirements during ICU and hospital stays, and longer lengths of stay than the other groups did (p < 0.001 in all analyses) ( Table 4 ). Moreover, the Severe Lymphopenia Group also had the highest mortality rate, not only in direct comparison but also in Kaplan‒Meier survival analysis (log-rank test p = 0.0048) ( Figure 2 ).

**Table 4:** Demographic characteristics, primary clinical characteristics and outcomes in the Nonlymphopenia, Lymphopenia > 500 cell/µL, < 1,000 cell/µL and < 500cell/µL groups

	Nonlymphopenia Group (n = 191; 20.9%)	Lymphopenia Group > 500 cell/uL and < 1,000 cell/uL (n = 398; 43.6%)	Lymphopenia Group ≤ 500 cell/uL (n = 323; 35.5%)	p value
Age (years)	60.7 ± 17.9	66.8 ± 15.9	66.7 ± 14.4	< 0.001
Sex (males)	108 (56.5)	251 (63)	235 (72.8)	0.001
ICU admission				
SOFA at admission	1 (0 - 2)	3 (1 - 4)	4 (2 -7)	< 0.001
SAPS III at admission	45.8 ± 13.1	47.7 ± 11.3	51.8 ± 12.9	< 0.001
Laboratory results				
Max registered C-reactive protein (mg/dL)	8.3 (3.4 - 19.6)	15.0 (5.9 - 21.4)	16.8 (7.9 - 24.1)	< 0.001
Max registered procalcitonin (ng/mL)	0.46 (0.14 - 0.77)	0.5 (0.16 - 0.9)	0.47 (0.18 - 0.9)	0.502
Minimum leucocytes count registered (x10^9^)	6.8 ± 3.7	5.6 ± 2.4	4.9 ± 2.4	< 0.001
Max troponin registered (ng/mL)	25 (8.5 - 56.7)	24 (12.7 - 76.5)	59 (12.7 - 132.5)	0.03
Outcomes				
Mechanical ventilation	42 (22)	81 (20.4)	118 (36.8)	< 0.001
Vasopressor support	38 (19.9)	78 (19.5)	117 (36.5)	< 0.001
Renal replacement therapy	16 (8.4)	37 (9.3)	59 (36.5)	< 0.001
ICU length of stay (days)	5 (3 - 10)	7 (4 - 12.8)	12 (7 - 22)	< 0.001
Hospital length of stay (days)	7 (3 ± 14)	9 (4 ± 17)	14 (8 ± 27)	< 0.001
Mortality	17 (8.9)	32 (8.0)	47 (14.6)	0.016

ICU - intensive care unit; SOFA - Sequential Organ Failure Assessment; SAPS - Simplified Acute Physiology Score. Results expressed as the mean ± standard deviation, n (%) or median (interquartile range).

ICU - intensive care unit.

## DISCUSSION

Our study revealed that lymphopenia is a biomarker of severity in COVID-19 patients, with a higher prevalence than previously acknowledged and noted in other studies.^( 2 , 3 , 13 , 16 )^ Our findings revealed that lymphopenia is associated with higher severity scores (SOFA and SAPS III), elevated levels of serum inflammatory biomarkers (CRP and PCT) at both admission and during the ICU, and a greater need for vasopressor support. Furthermore, patients with lymphopenia at ICU admission experience more severe COVID-19 and require longer stays in the ICU and hospital.

Our study highlights the significant impact of lymphopenia on the fatal outcome of COVID-19 patients admitted to the ICU, revealing an 11% greater risk of death even after adjusting for multiple variables through multivariable analysis. This finding aligns with previously published evidence showing an inverse correlation between lymphocyte count and adverse outcomes, such as organ dysfunction and in-hospital mortality.^( 4 , 5 , 6 , 23 )^ These results are further reinforced by the results of the multivariable logistic regression analysis after matching, revealing an even greater risk contribution of lymphopenia to mortality, with a higher odds ratio of 1.621 with the proposed covariables. These data reinforce previously hypothesized mechanisms linking lymphopenia to immunosuppression through T-cell exhaustion syndrome and uncontrolled proinflammatory effects, which may contribute to the abnormally high mortality risk observed in COVID-19 patients.^( 7 , 17 , 20 - 22 )^ Additionally, our findings demonstrate an association between lymphocyte count and poor outcomes in COVID-19 patients, corroborating key insights from prominent meta-analyses on this issue.^( 5 , 6 , 12 , 24 )^

Furthermore, interaction analysis expanded upon this impact, revealing a heightened mortality risk associated with lymphopenia, with no discernible interaction with other variables associated with mortality, such as the SOFA score at ICU admission or a history of COPD or CKD. Our analysis also revealed a significant association between age and lymphopenia, with an 8% increase in mortality for each decade of life in patients with concurrent lymphopenia. This effect appears to be particularly pronounced in patients aged 60-80 years, suggesting a heightened impact on mortality within this age range. This finding highlights the particular importance of lymphopenia in certain subsets of patients, considering the findings of previous studies indicating that, in addition to COVID‐19, there is an age‐associated reduction in total, central memory (CM), and early CD8+ T-cell subsets, as well as naïve and regulatory CD4+ T-cell subsets. Additionally, these patients exhibit a strong association between lymphocyte count and a composite poor outcome that it is significantly affected by age.^( 6 , 25 )^ Recognizing this subset of patients influenced by lymphopenia may have a profound clinical impact, underscoring the importance of maintaining high clinical suspicion and prioritizing early recognition and support for these individuals.

However, no significant differences in the Kaplan‒Meier survival analysis were observed between the Lymphopenia and Nonlymphopenia Groups, regardless of matching. These results suggest comparable all-cause in-hospital mortality rates, irrespective of the presence of lymphopenia, when the disease’s progression over time is considered, highlighting the intricate nature of COVID-19 and the likely influence of various factors stemming from its high inflammatory state, numerous complications, and prolonged ICU and hospital stays. These data contribute to explaining the discrepancies in evidence collected surrounding the use of lymphopenia as a biomarker in patients with COVID-19. While some studies have identified lymphopenia as an independent risk factor for mortality in hospitalized COVID-19 patients and advocate for its dynamic monitoring as a predictor of poor outcomes,^( 12 , 16 , 18 )^ these results are often due to differences in defining adverse outcomes, as highlighted in recent meta-analyses.^( 5 , 6 )^ Therefore, our findings suggest that while lymphopenia may directly impact mortality, its significance is heavily influenced by patient and disease factors, playing a pivotal role within a complex network of multivariable interactions throughout the disease course.

Our subanalysis, stratifying lymphopenia by severity, confirmed its clinical impact on COVID-19 patients. Notably, patients with more severe lymphopenia demonstrated a markedly greater demand for organ support and experienced prolonged hospital and ICU stays. Furthermore, in the survival analysis, patients with an absolute serum lymphocyte count ≤ 0.5 × 10^9^/L presented significantly elevated mortality rates compared with those with milder forms of lymphopenia upon ICU admission. These findings underscore the importance of considering lymphopenia severity when interpreting the results of survival analyses. This aligns with prior research suggesting that lymphopenia below the threshold of 0.5 × 10^9^/L may independently correlate with poor outcomes in this patient population^( 26 )^ and that severe lymphopenia is significantly associated with an increased likelihood of mortality, even among immunocompromised individuals.^( 27 )^ This clarification emphasizes that the influence of lymphopenia on previous results is significantly influenced by its severity, and it should serve as a robust clinical indicator, highlighting a subset of patients who may require tailored management and treatment strategies.

Future research should explore the underlying mechanisms linking lymphopenia to specific age ranges and severity levels, particularly in the context of evolving treatments. Such investigations are crucial for identifying potential interventions aimed at mitigating the adverse effects of lymphopenia and improving patient outcomes in the ever-changing landscape of COVID-19 management.

Our study has several strengths. It is grounded in a large cohort of critical care COVID-19 patients with prospectively collected data, with the participation of centers across multiple countries. This broad representation allows for a representative cohort of patients, strengthening the results presented and enhancing external validity. Additionally, it used a matching system with only a small number of patients excluded and a minimal rate of missing data to minimize potential individual and systematic bias.

However, we acknowledge several limitations in our study. This study focused primarily on in-hospital mortality, and the absence of follow-up may not capture longer-term outcomes and complications. Furthermore, it does not consider COVID-19 treatment strategies that have changed over the course of the pandemic, which could impact the assessment of mortality risk and matching. Despite our efforts to match patients using propensity scores, there may be unmeasured confounding variables that were potentially not considered in our analysis. These unaccounted factors might influence the observed associations.

## CONCLUSION

Our study highlights the prevalence of lymphopenia among critically ill COVID-19 patients and elucidates its independent associations with disease severity, elevated levels of serum inflammatory biomarkers (C-reactive protein and procalcitonin), and prolonged intensive care unit and hospital stays. Through propensity score matching, we further established the significant risk contribution of lymphopenia to mortality, with a notable odds ratio. Moreover, our interaction models revealed a significant association between age and lymphopenia, indicating an increase in mortality for each decade of longevity in patients with concurrent lymphopenia, thus positioning this marker as an independent variable in patient outcomes.

## SUPPLEMENTARY MATERIAL


