# Supplementary material for: Advancing insights in critical COVID-19: unraveling lymphopenia through propensity score matching - Findings from the Multicenter LYMPH-COVID Study
Source: Crit Care Sci. 2024 Sep 18;36:e20240236en. doi: 10.62675/2965-2774.20240236-en (PMC11463993; doi:10.62675/2965-2774.20240236-en)
Supplement: Supplementary file 1 [file 2965-2774-ccsci-36-e20240236en-suppl01.pdf]

*Critical Care Science*
https://doi.org/10.62675/2965-2774.20240236-en

SUPPLEMENTARY MATERIAL

# Advancing insights in critical COVID-19: unraveling lymphopenia through propensity score matching. Findings from the Multicenter LYMPH-COVID Study

José Pedro Cidade[1], Vicente Cês Souza-Dantas[2], Rafaela Braga Mamfrim[3], Renata Carnevale Miranda[3], Henrique Tommasi Caroli[3], Natália Almeida Oliveira[3], Alessandra Figueiredo Thompson[3], Gabriela E. Oliveira[3], Pedro Póvoa[1]

**Table 1S -** Cox regression model for mortality accounting for Lymphopenia Groups and co-variables found to be significantly associated with mortality during univariate analysis*

| Variable | Coefficient | Hazard ratio | Standard error | p valor |
|---|---|---|---|---|
| Age | 0.0586 | 1.0603 | 0.0114 | < 0.001 |
| Sex (male) | 0.1849 | 1.2031 | 0.2590 | 0.4854 |
| Lymphopenia Group | 0.0438 | 1.0448 | 0.3626 | 0.9038 |
| SAPS III at ICU admission | 0.0067 | 0.9934 | 0.0095 | 0.4834 |
| SOFA score at ICU admission | 0.2234 | 1.2504 | 0.0358 | < 0.001 |
| Chronic obstructive pulmonary disease | 0.5138 | 1.6717 | 0.3041 | 0.0911 |
| Chronic kidney disease | 0.3114 | 1.3654 | 0.3051 | 0.3074 |
| Obesity | 0.4016 | 1.4942 | 0.6052 | 0.1882 |
| Diabetes | 0.0403 | 1.0411 | 0.2485 | 0.8712 |

SAPS - Simplified Acute Physiology Score; ICU - intensive care unit; SOFA - Sequential Organ Failure Assessment. *Concordance of the model 0.866 (standard error = 0.019); Wald test $\chi^2 = 146.3$, df = 10, $p < 0.001$; Significant at the 0.05 level.

**Table 2S -** Multiple logistic regression Interaction models accounting for interaction modelling between age and lymphopenia*

| Variable | Coefficient | Odds ratio | Standard error | p valor |
|---|---|---|---|---|
| Age | 0.010 | 0.990 | 0.009 | 0.275 |
| Gender (male) | -1.020 | 0.360 | 0.300 | 0.1 |
| Lymphopenia Group | 0.655 | 0.001 | 2.783 | 0.019 |
| SOFA score at ICU admission | 0.224 | 1.277 | 0.037 | < 0.001 |
| Chronic obstructive pulmonary disease | 0.181 | 0.835 | 0.450 | 0.688 |
| Chronic kidney disease | 1.267 | 3.550 | 0.449 | 0.005 |
| Age: Lymphopenia Group | 0.077 | 1.080 | 0.034 | 0.025 |

SOFA - Sequential Organ Failure Assessment; ICU - intensive care unit. *Concordance of the model 0.866 (standard error = 0.019); Wald test $\chi^2 = 146.3$, df = 10, $p < 0.001$.


This is an open access article under the CC BY license (https://creativecommons.org/licenses/by/4.0/).

**Table 3S -** Demographic and primary clinical characteristics in the Nonlymphopenia and Lymphopenia Groups after propensity score matching

| | Nonlymphopenia Group (n = 153) | Lymphopenia Group (n = 153) | p valor |
|---|---|---|---|
| Age (years) | 58 (46 - 74) | 60 (50 - 74) | 0.495 |
| Gender (males) | 83 (54.2) | 74 (48.4%) | 0.360 |
| Admission | | | |
| SOFA at admission | 1 (0 - 2) | 1 (0 - 2) | 0.468 |
| SAPS III at admission | 44 (39 - 51) | 45 (41 - 50) | 0.254 |
| Clinical characteristics | | | |
| Chronic obstructive pulmonary disease | 4 (2.6) | 6 (3.9) | 0.748 |
| Chronic kidney disease | 7 (4.6) | 8 (5.2) | 1.000 |
| Asthma | 14 (9.2) | 17 (11.1) | 0.705 |
| Obesity | 29 (19) | 28 (18.3) | 1.000 |
| Diabetes | 31 (20.3) | 29 (19) | 0.886 |
| Heart disease | 23 (15) | 22 (14.4) | 1.000 |
| ICU length of stay | | | |
| Mechanical ventilation | 22 (14.4) | 25 (16.4) | 0.244 |
| Vasopressor support | 21 (13.7) | 14 (9.2) | 0.249 |
| Renal replacement therapy | 9 (5.9) | 8 (5.2) | 0.589 |
| ICU length of stay (days) | 7 (5 - 18) | 10 (7 - 14) | 0.194 |
| Hospital length of stay (days) | 12 (6 - 26) | 11 (8 - 19) | 0.769 |
| Mortality | 7 (4.6) | 10 (6.5) | 0.596 |

SOFA - Sequential Organ Failure Assessment; SAPS - Simplified Acute Physiology Score; ICU - intensive care unit. Results expressed as median (interquartile range) or n (%).

**Table 4S -** Standard mean deviation calculations of propensity score matching

| Covariate | Standard mean deviation | |
|---|---|---|
| | Before matching | After matching |
| Age | 0.0839 | 0.0000 |
| SAPS III | 0.1026 | 0.0000 |
| SOFA admission | 0.002 | 0.0000 |
| Inflammatory leucopenia | 0.4666 | 0.0000 |
| Chronic obstructive pulmonary disease | 0.9477 | 0.092 |
| Asthma | - 0.817 | 0.077 |
| Obesity | - 0.6209 | 0.063 |
| Chronic kidney disease | 0.9085 | 0.089 |
| Diabetes | 0.5948 | 0.062 |
| Heart disease | 0.6993 | 0.071 |

SAPS - Simplified Acute Physiology Score; ICU - intensive care unit; SOFA - Sequential Organ Failure Assessment.

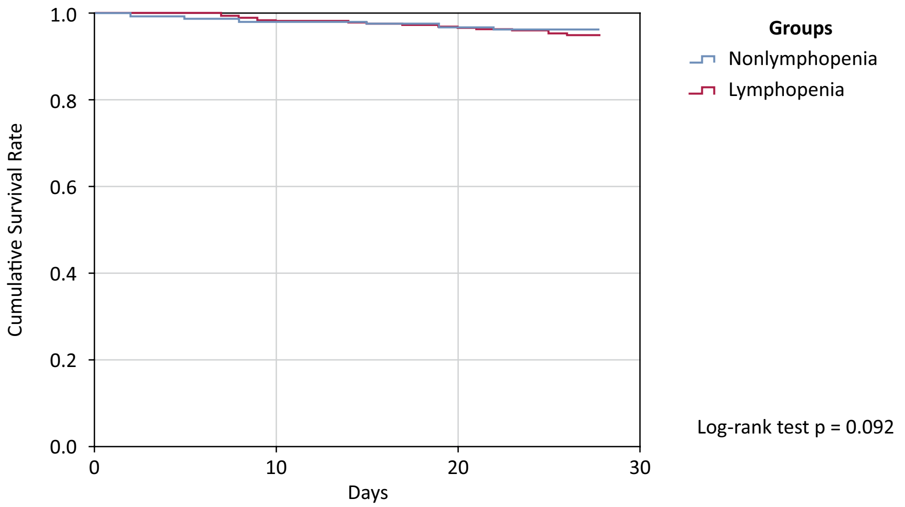


Number of in-risk patients

| Groups/Days | O | S | 10 | 15 | 20 | 25 | 28 |
|---|---|---|---|---|---|---|---|
| Nonlymphopenia | 191 | 187 | 181 | 181 | 178 | 174 | 174 |
| Lymphopenia | 721 | 714 | 700 | 689 | 675 | 650 | 542 |

**Figure 1S -** Kaplan-Meier survival analysis between unmatched Nonlymphopenia and Lymphopenia Groups.

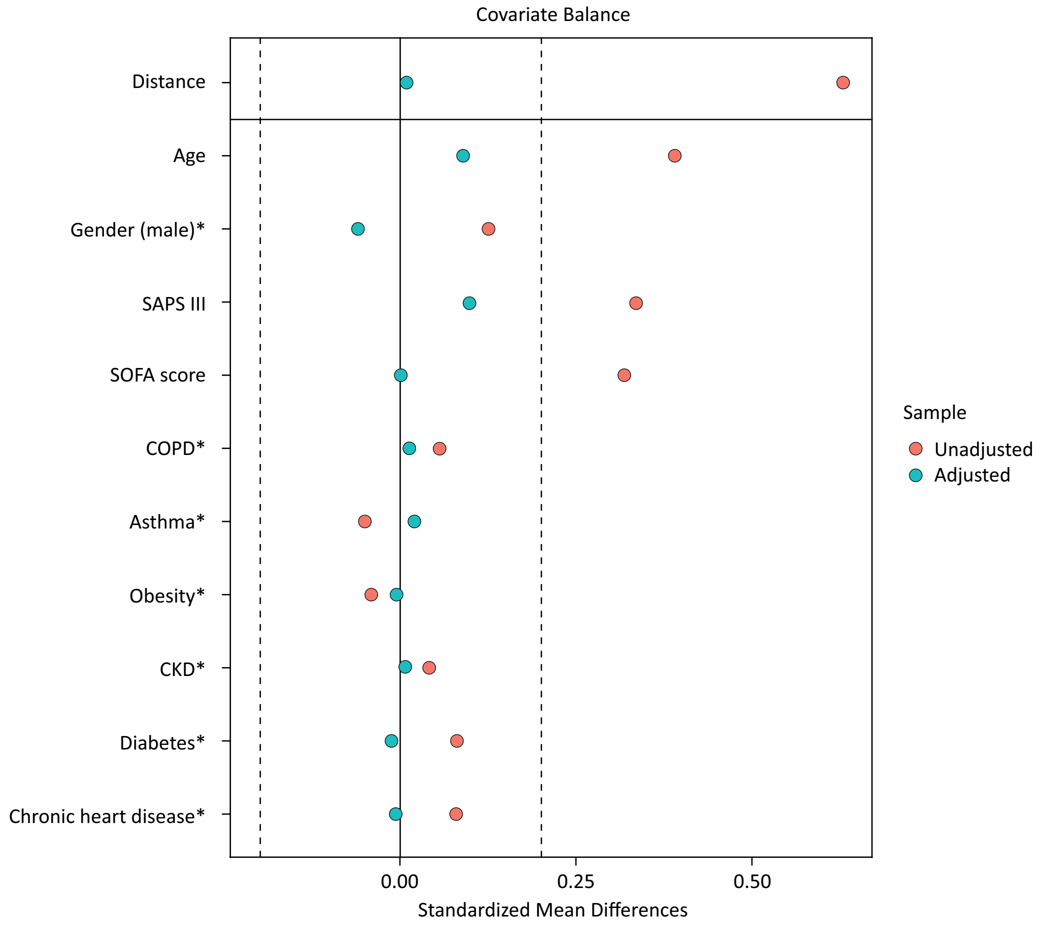


**Figure 2S -** Love plots after propensity score matching.
SAPS - Simplified Acute Physiology Score; SOFA - Sequential Organ Failure Assessment; COPD - chronic obstructive pulmonary disease; CKD - chronic kidney disease.

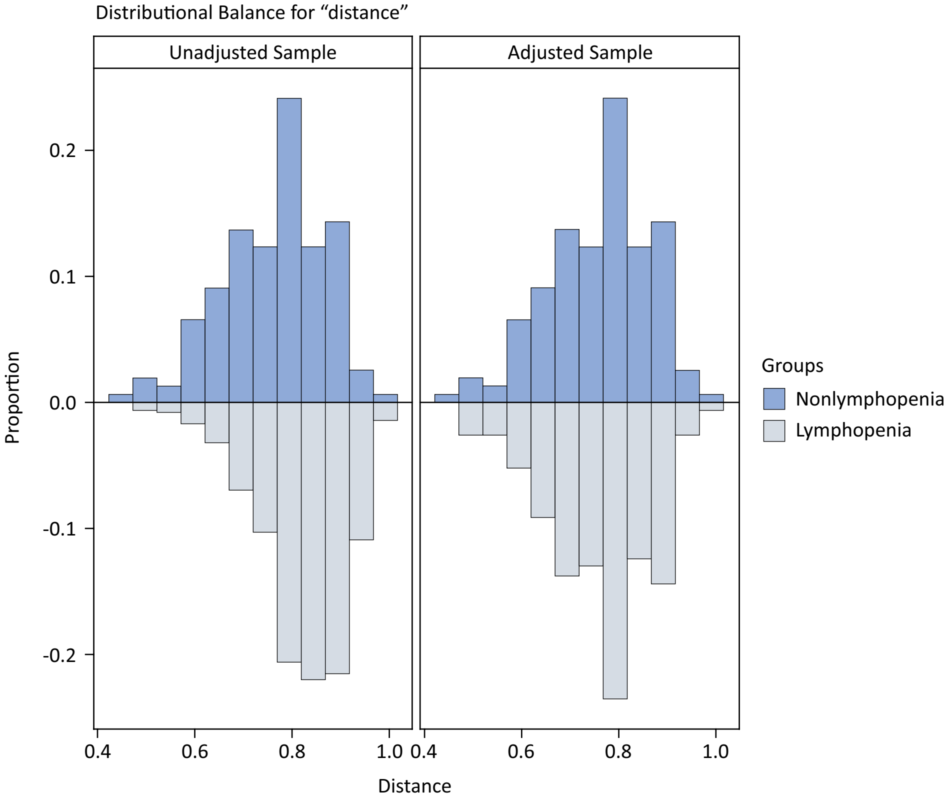


**Figure 3S -** Distributional balance after propensity score matching.

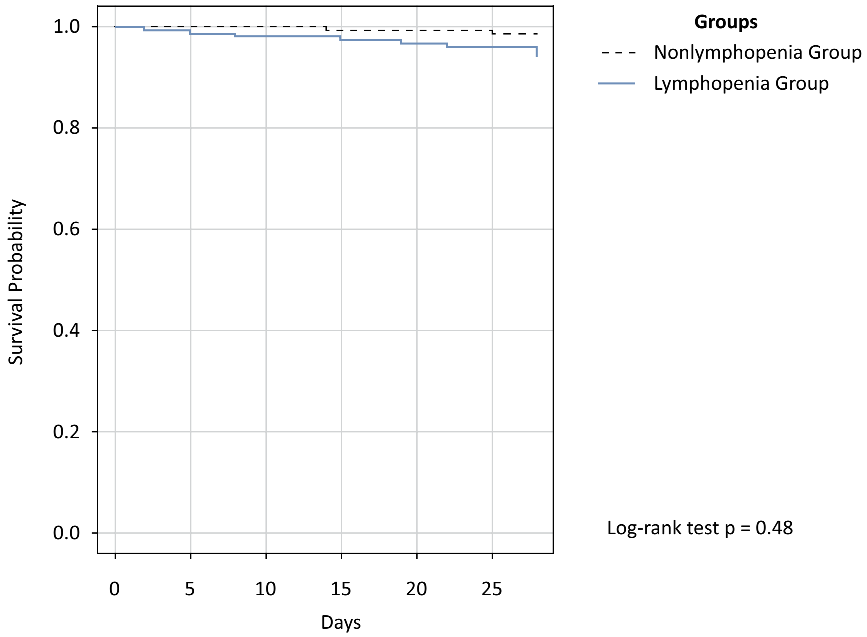


Number of in-risk patients

| Groups/Days | O | S | 10 | 15 | 20 | 25 | 28 |
|---|---|---|---|---|---|---|---|
| Nonlymphopenia | 153 | 153 | 153 | 149 | 148 | 146 | 146 |
| Lymphopenia | 153 | 151 | 148 | 147 | 145 | 143 | 143 |

**Figure 4S -** Kaplan-Meier survival analysis between matched Nonlymphopenia and Lymphopenia Groups.
