# Supplementary material for: Advancing insights in critical COVID-19: unraveling lymphopenia through propensity score matching - Findings from the Multicenter LYMPH-COVID Study
Source: Crit Care Sci. 2024 Sep 18;36:e20240236en. doi: 10.62675/2965-2774.20240236-en (PMC11463993; doi:10.62675/2965-2774.20240236-en)
Supplement: Translation

# Translation: Avanços na compreensão da COVID-19 grave: desvendar a linfopenia por meio da correspondência da pontuação de propensão – Resultados do estudo multicêntrico LYMPH-COVID

**DOI:** 10.62675/2965-2774.20240236-pt

**Authors:** José Pedro Cidade, Vicente Cês Souza-Dantas, Rafaela Braga Mamfrim, Renata Carnevale Miranda, Henrique Tommasi Caroli, Natália Almeida Oliveira, Alessandra e Figueiredo Thompson, Gabriela E. Oliveira, Pedro Póvoa

**Affiliations:** 1 Portugal Unidade de Terapia Intensiva 4, Departamento de Cuidados Intensivos, Hospital São Francisco Xavier, Centro Hospitalar Lisboa Ocidental - Lisboa, Portugal.; 2 Brasil Instituto D’Or de Pesquisa e Ensino - Rio de Janeiro (RJ), Brasil.; 3 Brasil Hospital Copa D’Or - Rio de Janeiro (RJ), Brasil.

**Keywords:** Linfopenia, COVID-19, Infecções por coronavírus, SARS-CoV-2, Mortalidade, Cuidados críticos, Unidades de terapia intensiva

## Abstract

**Objetivo:**

Elucidar o impacto da linfopenia nos desfechos críticos de pacientes com COVID-19.

**Métodos:**

Trata-se de estudo de coorte prospectivo multicêntrico realizado em cinco hospitais de Portugal e do Brasil entre 2020 e 2021. O estudo incluiu pacientes adultos internados na unidade de terapia intensiva com pneumonia por SARS-CoV-2. Os pacientes foram categorizados em dois grupos com base em suas contagens de linfócitos dentro de 48 horas da admissão na unidade de terapia intensiva: o Grupo Linfopenia (contagem sérica de linfócitos < 1 × 10^9^/L) e o Grupo sem Linfopenia. Foram utilizadas regressão logística multivariada, correspondência de escore de propensão, análise da curva de sobrevida de Kaplan-Meier e análise de regressão de riscos proporcionais de Cox.

**Resultados:**

Participaram 912 pacientes, sendo 191 (20,9%) no Grupo sem Linfopenia e 721 (79,1%) no Grupo com Linfopenia. Os pacientes com linfopenia apresentaram índices de gravidade da doença significativamente elevados, incluindo a *Sequential Organ Failure Assessment* e o *Simplified Acute Physiology Score 3* , na admissão na unidade de terapia intensiva (p = 0,001 e p < 0,001, respectivamente). Além disso, eles apresentaram maior necessidade de suporte vasopressor (p = 0,045) e internações prolongadas na unidade de terapia intensiva e no hospital (ambos p < 0,001). A análise de regressão logística multivariada após a correspondência da pontuação de propensão revelou significativa contribuição da linfopenia para a mortalidade, com razão de chances de 1.621 (IC95% 1.275 - 2.048; p < 0,001). Os modelos de interação revelaram aumento de 8% na mortalidade para cada década de longevidade em pacientes com linfopenia concomitante. Na subanálise que utilizou a estratificação em três grupos, o Grupo com Linfopenia grave apresentou a maior taxa de mortalidade, não apenas em comparações diretas, mas também na análise de sobrevida de Kaplan-Meier (teste *log-rank* p = 0,0048).

**Conclusão:**

A linfopenia em pacientes com COVID-19 está associada ao aumento da gravidade da doença e ao aumento do risco de mortalidade, ressaltando a necessidade de suporte imediato para pacientes críticos de alto risco. Esses achados oferecem informações importantes para melhorar as estratégias de assistência a pacientes com COVID-19.

## INTRODUÇÃO

A linfopenia é uma característica distinta observada em pacientes críticos com a doença do coronavírus 2019 (COVID-19) e supostamente serve como característica definitiva da doença.^( 1 )^Nos últimos anos, ela tem sido um marcador de interesse, motivando os pesquisadores a encontrarem evidências convincentes que apoiem seu potencial como um indicador específico ligado à gravidade da doença e às altas taxas de mortalidade.^( 2 - 5 )^A incidência de linfopenia entre os pacientes com COVID-19 internados no hospital foi relatada como sendo de 63%, aumentando para quase 85% entre aqueles em estado grave.^( 6 )^Isso ressalta sua posição única como um forte elo entre a resposta inflamatória desregulada e a produção de citocinas descrita na fisiopatologia da doença.^( 7 , 8 )^Desvendar os intrincados mecanismos subjacentes à linfopenia em pacientes com COVID-19 pode revelar uma nova influência direta do sistema imunológico e os desfechos de pacientes com COVID-19.

Vários biomarcadores clínicos e laboratoriais foram amplamente caracterizados, avaliando seu valor preditivo na identificação imediata de pacientes que necessitam de suporte multiorgânico e daqueles que enfrentam um alto risco de mortalidade.^( 9 , 10 )^A linfopenia induzida pela síndrome respiratória aguda grave do coronavírus 2 (SARS-CoV-2), que é atribuída à infecção e à replicação do vírus nas células imunológicas, culmina em apoptose e está fortemente correlacionada com a necessidade de internação em unidade de terapia intensiva (UTI) e desfechos desfavoráveis.^( 11 - 15 )^Além disso, evidências recentes mostraram que a linfopenia (definida como uma contagem absoluta de linfócitos no soro < 1,0 × 10^9^/L) na admissão na UTI está significativamente associada ao aumento da necessidade de ventilação mecânica, terapias de substituição renal e suporte vasopressor.^( 3 , 16 - 19 )^Por fim, metanálise recente posicionou a linfopenia como um biomarcador de avaliação essencial, indicando sua utilidade como marcador prognóstico em pacientes com COVID-19, particularmente entre indivíduos mais jovens. Essa associação levou a risco três vezes maior de desfechos desfavoráveis em pacientes hospitalizados com COVID-19 em outro estudo.^( 6 )^

No entanto, ainda faltam evidências para determinar se a associação observada entre linfopenia e mortalidade decorre diretamente da própria linfopenia ou se reflete principalmente a maior gravidade da infecção subjacente por COVID-19. Embora evidências substanciais tenham indicado correlação robusta entre linfopenia e desfechos adversos, é imperativo reconhecer a necessidade existente de elucidar o impacto independente da linfopenia no risco de mortalidade no contexto da COVID-19 e sua verdadeira utilidade como alvo de prognóstico nessa doença.^( 20 - 22 )^Para abordar essa questão, nosso estudo pretendeu avaliar o impacto da linfopenia nos desfechos críticos de pacientes com COVID-19.

## MÉTODOS

### Desenho do estudo e definição da coorte

Conduzimos um estudo de coorte observacional multicêntrico prospectivo em cinco UTIs do Brasil e de Portugal. Os Comitês de Ética do Hospital CopaStar e do Hospital Copa D’Or, no Rio de Janeiro, e a Comissão de Ética para Investigação Clínica em Lisboa aprovaram este estudo (CAAE: 17079119.7.0000.5249; e REC: 2020_EO_02, respectivamente).

Todos os pacientes adultos admitidos consecutivamente na UTI entre 1° de janeiro de 2020 e 31 de março de 2021 foram considerados para o estudo. Os pacientes foram incluídos se tivessem sido admitidos na UTI com diagnóstico de infecção respiratória por COVID-19 e período de internação de pelo menos 72 horas. A infecção respiratória por COVID-19 foi diagnosticada por meio de critérios clínicos e radiológicos que confirmaram o envolvimento pulmonar, com um teste de reação em cadeia da polimerase de transcrição reversa (RT-PCR) positivo para SARS-CoV-2.

Dentro da coorte do estudo, os pacientes foram estratificados em dois grupos distintos com base em suas contagens de linfócitos dentro de 48 horas após a admissão na UTI: o Grupo Linfopenia, composto de todos os pacientes com linfopenia documentada (contagem absoluta de linfócitos no soro < 1 × 10^9^/L), e o Grupo sem Linfopenia, que incluiu todos os pacientes sem diagnóstico de linfopenia.

Em subanálise diferente, os pacientes foram ainda estratificados em três grupos distintos (estratificação em três grupos) para avaliar o impacto do grau de linfopenia no desfecho primário: o Grupo Linfopenia Grave (contagem absoluta de linfócitos no soro ≤ 0,5 × 10^9^/L), o Grupo Linfopenia Não Grave (contagem absoluta de linfócitos no soro > 0,5 × 10^9^/L e < 1 × 10^9^/L) e o Grupo sem Linfopenia (contagem absoluta de linfócitos no soro ≥ 1 × 10^9^/L).

### Coleta e tratamento de dados

Os dados clínicos foram coletados prospectivamente dos registros eletrônicos de saúde dos pacientes e incluíram dados demográficos (idade e sexo), comorbidades (obesidade, tabagismo, hipertensão, *diabetes mellitus* , doença pulmonar obstrutiva crônica [DPOC], doença coronariana, doença renal crônica [DRC] e malignidade), resultados laboratoriais diários (contagem absoluta de linfócitos no soro, proteína C-reativa [PCR], procalcitonina [PCT] e troponina), *Simplified Acute Physiology Score III* (SAPS III), *Sequential Organ Failure Assessment* (SOFA), necessidades de suporte de órgãos (ventilação mecânica, suporte vasopressor e terapia de substituição renal) e dados de desfecho (tempo de internação na UTI e no hospital e taxas de mortalidade). Os dados foram armazenados em um banco de dados pseudoanonimizado.

O desfecho primário foi a taxa de mortalidade por todas as causas na UTI. Os desfechos secundários foram o tempo de permanência na UTI e no hospital e os requisitos de suporte de órgãos, incluindo suporte respiratório, hemodinâmico ou renal.

### Plano de análise estatística

#### Estatística descritiva e análise univariada

Na população analisada, foram realizadas análises univariadas e multivariadas para comparar os grupos com linfopenia e sem linfopenia não pareados. As variáveis contínuas são expressas como média e desvio-padrão (DP) para distribuições gaussianas e como mediana (intervalo interquartil [IIQ] para variáveis com distribuição não normal). As variáveis categóricas são apresentadas como números e porcentagens. A análise univariada foi realizada por meio do teste *t* de Student e do teste U de Mann-Whitney para variáveis contínuas e do teste do qui-quadrado para variáveis categóricas.

#### Análise de sobrevida

Para a investigação da sobrevida, foram geradas curvas de sobrevida de Kaplan-Meier para avaliar as taxas de mortalidade em 28 dias, complementadas com o respectivo teste *log-rank* . Os pacientes que receberam alta ou foram transferidos foram considerados observações censuradas na análise, garantindo análise abrangente dos desfechos do tipo tempo-até-evento. Nessa análise, determinamos que a ocorrência de riscos concorrentes era mínima. Foram produzidos modelos de regressão de Cox para o desfecho primário, levando em conta as covariáveis que foram significativamente associadas à mortalidade durante a análise univariada. As variáveis incluídas foram idade, sexo e comorbidades (obesidade, hipertensão, diabetes, DPOC, doença cardíaca crônica e DRC) e SAPS II e SOFA na admissão na UTI.

#### Análise de regressão logística multivariada

Realizou-se uma regressão logística multivariada para examinar de forma abrangente a influência das principais variáveis no desfecho primário. O limite de significância para inclusão de variáveis no modelo foi definido como p < 0,05, garantindo um critério de seleção rigoroso. As variáveis incorporadas ao modelo incluíram idade, sexo, presença de linfopenia na admissão na UTI, comorbidades e SAPS II e SOFA na admissão na UTI.

Além disso, os modelos de interação foram sistematicamente construídos para estimar os possíveis impactos cumulativos das variáveis independentes. Seguindo os resultados obtidos em nossa análise anterior, em que a linfopenia, a idade e o SOFA na admissão apresentaram associações significativas, exploramos especificamente as interações entre essas variáveis. A seleção do melhor modelo comparativo foi determinada pela identificação do modelo com o menor critério de informação de Akaike (AIC).

#### Correspondência de escore de propensão

Para criar grupos de estudo comparáveis com e sem linfopenia, empregou-se a correspondência de escore de propensão de 1 para 1 com vizinhança ideal. As pontuações de propensão foram obtidas por meio de modelagem de regressão logística, incorporando 10 critérios de correspondência - idade, sexo, obesidade, hipertensão, diabetes, DPOC, doença cardíaca crônica, DRC e pontuações SAPS II e SOFA na admissão na UTI – usando o pacote MatchIt no R. Foram aplicados calibradores, com largura especificada de 0,05, durante a correspondência do escore de propensão para aumentar o equilíbrio entre os grupos.

Para avaliar a eficácia do procedimento de correspondência, foram examinados minuciosamente os diagnósticos de equilíbrio, incluindo *love plots* , gráficos de distribuição de escore de propensão e diferenças médias padronizadas. As diferenças médias padronizadas foram calculadas para avaliar o equilíbrio alcançado após a correspondência, assegurando que os grupos pareados fossem comparáveis em termos de covariáveis basais.

#### Tratamento de dados ausentes

Os pacientes cujos dados excederam 5% das variáveis analisadas ou cuja contagem absoluta de linfócitos séricos na admissão na UTI estava ausente foram excluídos da análise; adotou-se abordagem conservadora para manter a integridade dos dados e evitar possíveis vieses. Os dados ausentes abaixo dessa taxa foram imputados usando os valores médios das variáveis para minimizar o viés sistemático e manter a representatividade do conjunto de dados. Essa abordagem nos permitiu preservar a validade interna de nossas análises.

Todos os cálculos foram realizados por meio da interface *Statistical Package for the Social Sciences* (SPSS), versão 26.0.0.0, e do R, versão 4.0.3. Valores de p < 0,05 foram considerados estatisticamente significativos.

## RESULTADOS

Após a identificação, 973 pacientes foram inicialmente considerados aptos para o estudo. Entre esses pacientes, 61 foram excluídos da análise estatística, 35 foram excluídos porque não permaneceram na UTI por 72 horas ou mais e 26 foram excluídos porque mais de 5% das variáveis analisadas apresentavam dados ausentes. Os 912 pacientes restantes foram incluídos, conforme ilustrado na figura 1 . No Grupo sem Linfopenia, foram incluídos 91 pacientes (20,9%); no Grupo com Linfopenia, 721 pacientes (79,1%) apresentaram linfopenia na admissão, com contagem absoluta de linfócitos no soro < 1 × 10^9^/L. A tabela 1 resume as características demográficas basais e clínicas primárias dos pacientes.

**Figura 1:** Seleção dos participantes do estudo.

UTI - unidade de terapia intensiva.

**Tabela 1:** Dados demográficos, características clínicas primárias e desfechos nos grupos sem linfopenia e com linfopenia

	Grupo sem Linfopenia (n = 191; 20,9%)	Grupo Linfopenia (n = 721; 79,1%)	Valor de p
Idade (anos)	60,7 ± 17,9	66,6 ± 15,4	< 0,001
Sexo (masculino)	108 (56,5)	484 (67,1)	0,004
Comorbidades			
DPOC	5 (2,6)	57 (7,9)	0,067
Asma	16 (8,4)	29 (4)	0,058
Doença renal crônica	13 (6,8)	62 (8,6)	0,12
Obesidade	37 (19,4)	110 (15,3)	0,486
*Diabetes mellitus*	40 (20,9)	200 (27,7)	0,145
Doença cardíaca	31 (16,2)	172 (23,9)	0,001
Internação em UTI			
Proteína C-reativa (mg/dL)	6,4 (2,9 - 14,9)	7,4 (4,7 - 16,5)	0,005
Procalcitonina (ng/mL)	0,1 (0,07 - 0,17)	0,12 (0,08 - 0,29)	0,005
SOFA na admissão	1 (0 - 2)	4 (2 - 5)	< 0,001
SAPS III na admissão	45,8 ± 13,1	49,5 ± 12,2	< 0,001
Resultados laboratoriais			
Proteína C-reativa máxima registrada (mg/dL)	8,3 (3,4 - 19,6)	15,5 (6,7 - 23,1)	< 0,001
Procalcitonina máxima registrada (ng/mL)	0,46 (0,14 - 0,77)	0,5 (0,17 - 0,9)	0,286
Contagens mínimas de leucócitos registradas (x10^9^)	6,8 ± 3,7	5,32 ± 2,4	< 0,001
Troponina máxima registrada (ng/mL)	25 (8,5 - 56,7)	38 (13 - 111)	0,099
Desfechos			
Ventilação mecânica	42 (22)	199 (27,6)	0,229
Suporte com vasopressor	38 (19,9)	195 (27)	0,045
Terapia de substituição renal	16 (8,4)	96 (13,3)	0,054
Tempo de internação na UTI (dias)	5 (3 - 10)	9 (5 - 16,5)	< 0,001
Tempo de internação hospitalar (dias)	8 (3 ± 14)	10 (6 ± 22)	< 0,001
Mortalidade	17 (8,9)	79 (11)	0,621

DPOC - doença pulmonar obstrutiva crônica; UTI - unidade de terapia intensiva; SOFA - *Sequential Organ Failure Assessment* ; SAPS - *Simplified Acute Physiology Score* . Resultados expressos como média ± desvio-padrão, n (%) ou mediana (intervalo interquartil).

Os pacientes do Grupo com Linfopenia eram significativamente mais velhos e tinham maior probabilidade de serem do sexo masculino do que os do Grupo sem Linfopenia. Esses pacientes também apresentaram SOFA e SAPS III mais altos na admissão na UTI (p < 0,001 em ambas as análises) e níveis significativamente mais altos de biomarcadores inflamatórios séricos (PCR e PCT), tanto na admissão quanto na internação na UTI. Além disso, os pacientes que apresentaram linfopenia tiveram taxa maior de necessidade de suporte vasopressor, embora os grupos não tenham diferido em termos de terapias de substituição respiratória ou renal.

Com relação à análise dos desfechos, as taxas de mortalidade na UTI não diferiram nos dois grupos. Esse resultado também foi sustentado pela análise de sobrevida de Kaplan-Meier em ambos os grupos, com teste *log-rank* de p = 0,092 (Figura 1S - Material Suplementar). No entanto, os pacientes do Grupo com Linfopenia passaram mais tempo na UTI e no hospital (p < 0,001, em ambas as análises). De forma consistente, o modelo de regressão de Cox, incorporando variáveis significativamente associadas à mortalidade, não demonstrou associação estatisticamente significativa entre o Grupo com Linfopenia e a mortalidade (razão de risco de 1,0448; IC95% 0,5133 - 2,127), conforme ilustrado na tabela 1S (Material Suplementar).

A análise de regressão logística multivariada revelou uma contribuição de risco significativa da linfopenia na mortalidade, com razão de chances de 1,110 (IC95% 1,022 - 1,206; p = 0,011), concomitante à idade, à SOFA na admissão na UTI e ao histórico médico anterior de DPOC e DRC, conforme demonstrado na tabela 2 . Além disso, os modelos de interação revelaram associação significativa entre idade e linfopenia, com aumento de 8% na mortalidade a cada década de longevidade em pacientes com linfopenia concomitante (razão de chances de 1,080; p = 0,025) (Tabela 2S, Material Suplementar). A linearidade do coeficiente de interação foi confirmada pelo teste de Box-Tidwell (-7,55x10^-6^; p = 0,99). Não houve interação significativa entre linfopenia e SOFA na admissão (p = 0,17), histórico prévio de DPOC (p = 0,32) ou histórico prévio de DRC (p = 0,07), embora o coeficiente estimado sugira possível tendência de aumento do risco de mortalidade entre pacientes com linfopenia e SOFA mais altos.

**Tabela 2:** Resultados da regressão logística multivariável na estimativa dos possíveis impactos cumulativos das variáveis independentes na mortalidade*

	Razão de chances	IC95%	Valor de p
Idade	1,070	1,043 - 1,098	< 0,001
Sexo (masculino)	1, 147	0,768 - 2,159	0,601
SAPS III na admissão na UTI	1,008	0,984 - 1,032	0,493
Escore SOFA na admissão	1,248	1,161 - 1,340	< 0,001
Linfopenia	1,110	1,022 - 1,206	0,011
Doença pulmonar obstrutiva crônica	2,160	1,032 - 4,524	0,043
Doença renal crônica	3,385	1,881 - 6,104	< 0,001
Obesidade	1,808	0,960 - 3,404	0,079
*Diabetes mellitus*	0,764	0,481 - 1,212	0,315
Insuficiência cardíaca crônica	1,299	0,762 - 2,215	0,313

IC95% - intervalo de confiança de 95%; SAPS - *Simplified Acute Physiology Score* ; UTI - unidade de terapia intensiva; SOFA - *Sequential Organ Failure Assessment* . * Critério de informação de Akaike 501.

Foram comparados 306 pacientes entre os grupos mediante correspondência de escore de propensão, enquanto as variáveis mencionadas anteriormente foram consideradas para correspondência. As principais características demográficas e a análise do equilíbrio da distribuição do escore de propensão (usando cálculos de desvio-padrão, *love plots* e gráficos de distribuição) foram incluídas no Material Suplementar (Tabelas 3S e 4S e Figuras 2S e 3S). A análise de regressão logística multivariável após a correspondência revelou contribuição de risco significativa da linfopenia na mortalidade, com razão de chances maior que 1,621 (IC95% 1,275 - 2,048; p < 0,001) com as covariáveis indicadas no modelo proposto ( Tabela 3 ). A análise de sobrevida de Kaplan-Meier não revelou diferença na sobrevida ao longo do tempo entre os grupos com e sem linfopenia (teste *log-rank* p = 0,48) (Figura 4S - Material Suplementar).

**Tabela 3:** Resultados da regressão logística multivariável na estimativa dos possíveis impactos cumulativos das variáveis independentes sobre a mortalidade após a correspondência do escore de propensão*

	Razão de chances	IC95%	Valor de p
Idade	1,035	1,010 - 1,060	0,005
Sexo (masculino)	0,285	0,167 - 0,485	< 0,001
SAPS III na admissão na UTI	0,998	0,976 - 1,021	0,914
Escore SOFA na admissão	1,438	1,290 - 1,608	< 0,001
Linfopenia	1,621	1,275 - 2,048	<0,001
Doença pulmonar obstrutiva crônica	2,214	0,853 - 5,753	0,112
Doença renal crônica	8,190	3,166 - 7,215	< 0,001
Obesidade	2,706	1,839 - 3,594	< 0,001
*Diabetes mellitus*	1,955	0,905 - 2,235	0,08
Insuficiência cardíaca crônica	1,006	0,946 - 1,069	0,295

IC95% - intervalo de confiança de 95%; SAPS - *Simplified Acute Physiology Score* ; UTI - unidade de terapia intensiva; SOFA - *Sequential Organ Failure Assessment* . * Critério de informação de Akaike 408.

Na subanálise que utilizou a estratificação em três grupos, o Grupo com Linfopenia grave apresentou escores de disfunção orgânica significativamente mais altos na admissão na UTI, maior necessidade de suporte de órgãos durante a internação na UTI e no hospital e maior tempo de internação do que os outros grupos (p < 0,001 em todas as análises) ( Tabela 4 ). Além disso, o Grupo com Linfopenia grave também apresentou a maior taxa de mortalidade, não apenas na comparação direta, mas também na análise de sobrevida de Kaplan-Meier (teste *log-rank* p = 0,0048) ( Figura 2 ).

**Tabela 4:** Características demográficas, características clínicas primárias e desfechos nos grupos sem linfopenia, com linfopenia > 500 células/µL, < 1.000 células/µL e < 500 células/µL

	Grupo sem Linfopenia (n = 191; 20,9%)	Grupo Linfopenia > 500 células/uL e < 1.000 células/uL (n = 398; 43,6%)	Grupo Linfopenia ≤ 500 células/uL (n = 323; 35,5%)	Valor de p
Idade (anos)	60,7 ± 17,9	66,8 ± 15,9	66,7 ± 14,4	< 0,001
Sexo (masculino)	108 (56,5)	251 (63)	235 (72,8)	0,001
Admissão na UTI				
SOFA na admissão	1 (0 - 2)	3 (1 - 4)	4 (2 -7)	< 0,001
SAPS III na admissão	45,8 ± 13,1	47,7 ± 11,3	51,8 ± 12,9	< 0,001
Resultados laboratoriais				
Proteína C-reativa máxima registrada (mg/dL)	8,3 (3,4 - 19,6)	15,0 (5,9 - 21,4)	16,8 (7,9 - 24,1)	< 0,001
Procalcitonina máxima registrada (ng/mL)	0,46 (0,14 - 0,77)	0,5 (0,16 - 0,9)	0,47 (0,18 - 0,9)	0,502
Contagens mínimas de leucócitos registradas (x10^9^)	6,8 ± 3,7	5,6 ± 2,4	4,9 ± 2,4	< 0,001
Troponina máxima registrada (ng/mL)	25 (8,5 - 56,7)	24 (12,7 - 76,5)	59 (12,7 - 132,5)	0,03
Desfechos				
Ventilação mecânica	42 (22)	81 (20,4)	118 (36,8)	< 0,001
Suporte com vasopressor	38 (19,9)	78 (19,5)	117 (36,5)	< 0,001
Terapia de substituição renal	16 (8,4)	37 (9,3)	59 (36,5)	< 0,001
Tempo de internação na UTI (dias)	5 (3 - 10)	7 (4 - 12,8)	12 (7 - 22)	< 0,001
Tempo de internação hospitalar (dias)	7 (3 ± 14)	9 (4 ± 17)	14 (8 ± 27)	< 0,001
Mortalidade	17 (8,9)	32 (8,0)	47 (14,6)	0,016

UTI - unidade de terapia intensiva; SOFA - *Sequential Organ Failure Assessment* ; SAPS - *Simplified Acute Physiology Score* . Resultados expressos como média ± desvio-padrão, n (%) ou mediana (intervalo interquartil).

**Figura 2:** Análise de sobrevida de Kaplan-Meier na subanálise com estratificação em três grupos (Grupo sem Linfopenia, Grupo com Linfopenia não Grave e Grupo com Linfopenia Grave).

## DISCUSSÃO

Nosso estudo revelou que a linfopenia é um biomarcador de gravidade em pacientes com COVID-19, com prevalência mais alta do que a reconhecida anteriormente e observada em outros estudos.^( 2 , 3 , 13 , 16 )^Nossos achados revelaram que a linfopenia está associada a escores de gravidade mais altos (SOFA e SAPS III), níveis elevados de biomarcadores inflamatórios séricos (PCR e PCT) na admissão e durante a UTI e maior necessidade de suporte vasopressor. Além disso, os pacientes com linfopenia na admissão na UTI apresentam COVID-19 mais grave e exigem internações mais longas na UTI e no hospital.

Nosso estudo destaca o impacto significativo da linfopenia no desfecho fatal de pacientes com COVID-19 internados na UTI, revelando risco 11% maior de morte, mesmo após o ajuste de diversas variáveis por meio de análise multivariável. Esse achado está alinhado com evidências publicadas anteriormente que demonstram correlação inversa entre a contagem de linfócitos e desfechos adversos, como disfunção orgânica e mortalidade hospitalar.^( 4 , 5 , 6 , 23 )^Esses resultados são ainda mais reforçados pelos resultados da análise de regressão logística multivariável após a correspondência, revelando contribuição de risco ainda maior da linfopenia na mortalidade, com razão de chances maior que 1,621 com as covariáveis propostas. Esses dados reforçam mecanismos previamente hipotetizados que ligam a linfopenia à imunossupressão por meio da síndrome de exaustão de células T e efeitos pró-inflamatórios descontrolados, o que pode contribuir para o risco de mortalidade anormalmente alto observado em pacientes com COVID-19.^( 7 , 17 , 20 - 22 )^Além disso, nossos achados demonstram associação entre a contagem de linfócitos e desfechos desfavoráveis em pacientes com COVID-19, corroborando as principais percepções de metanálises importantes sobre essa questão.^( 5 , 6 , 12 , 24 )^

Além disso, a análise de interação ampliou esse impacto, revelando maior risco de mortalidade associado à linfopenia, sem interação perceptível com outras variáveis associadas à mortalidade, como o SOFA na admissão na UTI ou um histórico de DPOC ou DRC. Nossa análise também revelou associação significativa entre idade e linfopenia, com aumento de 8% na mortalidade para cada década de vida em pacientes com linfopenia concomitante. Esse efeito parece ser particularmente acentuado em pacientes com idade entre 60 e 80 anos, sugerindo impacto maior na mortalidade nessa faixa etária. Esse achado destaca a importância particular da linfopenia em determinados subgrupos de pacientes, considerando os achados de estudos anteriores indicativos de que, além da COVID-19, há uma redução associada à idade nos subgrupos de células T CD8+ totais, de memória central (MC) e precoces, bem como nos subgrupos de células T CD4+ ingênuas e reguladoras. Além disso, esses pacientes apresentam forte associação entre a contagem de linfócitos e um desfecho desfavorável composto, que é significativamente afetado pela idade.^( 6 , 25 )^Reconhecer esse subconjunto de pacientes influenciados pela linfopenia pode ter um impacto clínico profundo, ressaltando a importância de manter uma alta suspeita clínica e priorizar o reconhecimento e o apoio precoce a esses indivíduos.

No entanto, não foram observadas diferenças significativas na análise de sobrevida de Kaplan-Meier entre os grupos com linfopenia e sem linfopenia, independentemente da correspondência. Esses resultados sugerem taxas comparáveis de mortalidade hospitalar por todas as causas, independentemente da presença de linfopenia, quando se considera a progressão da doença ao longo do tempo, destacando a natureza intrincada da COVID-19 e a provável influência de vários fatores decorrentes de seu alto estado inflamatório, inúmeras complicações e internações prolongadas em UTIs e hospitais. Esses dados contribuem para explicar as discrepâncias nas evidências coletadas em torno do uso da linfopenia como biomarcador em pacientes com COVID-19. Embora alguns estudos tenham identificado a linfopenia como fator de risco independente para a mortalidade em pacientes hospitalizados com COVID-19 e defendam seu monitoramento dinâmico como um preditor de desfechos desfavoráveis,^( 12 , 16 , 18 )^esses resultados geralmente se devem a diferenças na definição de desfechos adversos, conforme destacado em metanálises recentes.^( 5 , 6 )^Portanto, nossos achados sugerem que, embora a linfopenia possa afetar diretamente a mortalidade, sua importância é fortemente influenciada por fatores do paciente e da doença, desempenhando papel fundamental em uma rede complexa de interações multivariáveis ao longo do curso da doença.

Nossa subanálise, ao estratificar a linfopenia por gravidade, confirmou seu impacto clínico nos pacientes com COVID-19. Notavelmente, os pacientes com linfopenia mais grave demonstraram demanda marcadamente maior por suporte de órgãos e tiveram internações prolongadas no hospital e na UTI. Além disso, na análise de sobrevida, os pacientes com uma contagem absoluta de linfócitos séricos ≤ 0,5 × 10^9^/L apresentaram taxas de mortalidade significativamente elevadas em comparação com aqueles com formas mais leves de linfopenia na admissão na UTI. Esses achados ressaltam a importância de considerar a gravidade da linfopenia ao interpretar os resultados das análises de sobrevivência. Isso alinha-se com investigações anteriores, que sugerem que a linfopenia abaixo do limiar de 0,5 × 10^9^/L pode correlacionar-se independentemente com desfechos desfavoráveis nessa população de pacientes^( 26 )^e que a linfopenia grave está significativamente associada a um aumento da probabilidade de mortalidade, mesmo entre indivíduos imunocomprometidos.^( 27 )^Esse esclarecimento enfatiza que a influência da linfopenia em resultados anteriores é significativamente influenciada por sua gravidade e deve servir como um indicador clínico robusto, destacando um subconjunto de pacientes que podem exigir estratégias de manejo e tratamento adaptadas.

Estudos futuros devem explorar os mecanismos subjacentes que ligam a linfopenia a faixas etárias e níveis de gravidade específicos, particularmente no contexto da evolução dos tratamentos. Tais investigações são cruciais para identificar potenciais intervenções destinadas a mitigar os efeitos adversos da linfopenia e a melhorar os desfechos de pacientes em um cenário em constante mudança de manejo da COVID-19.

O nosso estudo tem vários pontos fortes. Baseia-se numa grande coorte de pacientes com COVID-19 em terapia intensiva com dados coletados prospectivamente, com a participação de centros de diversos países. Essa ampla representação permite uma coorte representativa de pacientes, fortalecendo os resultados apresentados e aumentando a validade externa. Além disso, utilizou um sistema de correspondência com apenas um pequeno número de doentes excluídos e uma taxa mínima de dados ausentes para minimizar potenciais vieses individuais e sistemáticos.

No entanto, reconhecemos várias limitações em nosso estudo. Este estudo se concentrou principalmente na mortalidade hospitalar, e a ausência de seguimento pode não capturar resultados e complicações de longo prazo. Além disso, ele não considera as estratégias de tratamento da COVID-19 que mudaram ao longo da pandemia, o que poderia afetar a avaliação do risco de mortalidade e a correspondência. Apesar de nossos esforços em combinar pacientes usando escores de propensão, pode haver variáveis de confusão não mensuradas que potencialmente não foram consideradas em nossa análise. Esses fatores não considerados podem influenciar as associações observadas.

## CONCLUSÃO

Nosso estudo destaca a prevalência de linfopenia em pacientes com COVID-19 em estado crítico e elucida suas associações independentes com a gravidade da doença, níveis elevados de biomarcadores inflamatórios séricos (proteína C-reativa e procalcitonina) e internação prolongada em unidades de terapia intensiva e hospitalar. Por meio da correspondência de escore de propensão, estabelecemos ainda mais a contribuição de risco significativa da linfopenia na mortalidade, com notável razão de chances. Além disso, nossos modelos de interação revelaram associação significativa entre idade e linfopenia, indicando um aumento na mortalidade para cada década de longevidade em pacientes com linfopenia concomitante e posicionando esse marcador como uma variável independente nos desfechos dos pacientes.

## MATERIAL SUPLEMENTAR
